# Prediction of early neurologic deterioration in patients with perforating artery territory infarction using machine learning: a retrospective study

**DOI:** 10.3389/fneur.2024.1368902

**Published:** 2024-05-22

**Authors:** Wei Liu, Longbin Jia, Lina Xu, Fengbing Yang, Zixuan Guo, Jinna Li, Dandan Zhang, Yan Liu, Han Xiang, Hongjiang Cheng, Jing Hou, Shifang Li, Huimin Li

**Affiliations:** ^1^Department of Neurology, Jincheng People's Hospital, Jincheng, China; ^2^The First Clinical College of Changzhi Medical College, Changzhi, China

**Keywords:** early neurologic deterioration, perforating artery territory infarction, machine learning, prediction, apolipoprotein B

## Abstract

**Background:**

Early neurological deterioration (END) is a frequent complication in patients with perforating artery territory infarction (PAI), leading to poorer outcomes. Therefore, we aimed to apply machine learning (ML) algorithms to predict the occurrence of END in PAI and investigate related risk factors.

**Methods:**

This retrospective study analyzed a cohort of PAI patients, excluding those with severe stenosis of the parent artery. We included demographic characteristics, clinical features, laboratory data, and imaging variables. Recursive feature elimination with cross-validation (RFECV) was performed to identify critical features. Seven ML algorithms, namely logistic regression, random forest, adaptive boosting, gradient boosting decision tree, histogram-based gradient boosting, extreme gradient boosting, and category boosting, were developed to predict END in PAI patients using these critical features. We compared the accuracy of these models in predicting outcomes. Additionally, SHapley Additive exPlanations (SHAP) values were introduced to interpret the optimal model and assess the significance of input features.

**Results:**

The study enrolled 1,020 PAI patients with a mean age of 60.46 (range 49.11–71.81) years. Of these, 30.39% were women, and 129 (12.65%) experienced END. RFECV selected 13 critical features, including blood urea nitrogen (BUN), total cholesterol (TC), low-density-lipoprotein cholesterol (LDL-C), apolipoprotein B (apoB), atrial fibrillation, loading dual antiplatelet therapy (DAPT), single antiplatelet therapy (SAPT), argatroban, the basal ganglia, the thalamus, the posterior choroidal arteries, maximal axial infarct diameter (measured at < 15 mm), and stroke subtype. The gradient-boosting decision tree had the highest area under the curve (0.914) among the seven ML algorithms. The SHAP analysis identified apoB as the most significant variable for END.

**Conclusion:**

Our results suggest that ML algorithms, especially the gradient-boosting decision tree, are effective in predicting the occurrence of END in PAI patients.

## 1 Introduction

Perforating artery territory infarction (PAI), a subtype of single subcortical infarction (SSI) caused by the occlusion of a perforating artery, is frequently observed in acute ischemic stroke, accounting for approximately 15.3–25% of all stroke cases (1, 2). The etiology of PAI may involve several mechanisms, such as lipohyalinosis, large plaques in the parent artery, and microatheroma (3). Lipohyalinosis is a vasculopathy that affects cerebral small vessels, leading to a “lacunar infarct” (LI), a major contributor to PAI (4, 5). Similarly, large plaques in the parent artery with severe stenosis may result in perforating artery embolisms. Additionally, microatheromas may give rise to branch atheromatous disease (BAD), characterized by ischemic lesions of ≥15 mm in diameter, typically observed on radiological imaging and the absence of severe stenosis of the parent artery (6).

Early neurological deterioration (END), characterized as a rapid exacerbation of neurological symptoms during the acute phase of a stroke, has been observed in 20–43% of PAI patients (7–9) and is linked to unfavorable patient outcomes (10, 11). The efficacy of treatment strategies for END in PAI patients, particularly those without severe stenosis of the parent artery, such as in LI and BAD cases, remains uncertain. The prediction of END in these patients is complex and heterogeneous, posing challenges to clinical management. Thus, identifying risk factors, pinpointing high-risk patients, and implementing timely interventions are essential for managing END in PAI patients without severe stenosis of the parent artery.

Advancements in computing power, the proliferation of big data, and the evolution of algorithms have significantly propelled machine learning (ML) in disease prediction (12). ML algorithms, a crucial aspect of artificial intelligence, excel at discerning patterns within intricate datasets using computational methods (13, 14). Compared to traditional statistics, ML shows greater proficiency in forecasting complex clinical events influenced by numerous factors and variables (15). Consequently, this research aims to develop ML models adept at predicting END in PAI patients without severe stenosis of the parent artery, utilizing data from a real-world, single-center cohort database.

## 2 Methods

### 2.1 Study design and patients

This retrospective, observational study was conducted on a cohort of PAI patients at Jincheng People's Hospital from September 2016 to July 2022. The inclusion criteria were as follows: patients aged 18 years or older, those diagnosed with PAI, and those admitted within 24 h of symptom onset. PAI was characterized as a single, small subcortical infarction in the territory of a perforating arteriole (16), identified by magnetic resonance imaging (MRI), and without significant large vessel stenosis (>50%), as confirmed by magnetic resonance angiography (MRA) or computed tomography angiography (CTA), with no maximum diameter limit (17). The exclusion criteria encompassed patients with multiple or cortical lesions, a premorbid modified Rankin Scale (mRS) score of ≥2, stroke mimics, or MRI-negative stroke. The study was approved by the ethics committee of Jincheng People's Hospital, and written informed consent was waived due to its retrospective nature; all patient information was anonymized before analysis.

### 2.2 Baseline data

We collected baseline data, including demographic details such as age, sex, and body mass index (BMI). We also gathered information on current smoking and drinking habits (≥20 g/day), medical history (including stroke, hypertension, diabetes, coronary atherosclerotic heart disease, and atrial fibrillation), secondary prevention treatment, laboratory data, clinically significant features [such as time from onset to presentation, National Institutes of Health Stroke Scale (NIHSS) score, pre-stroke mRS score], acute phase treatment [including IV thrombolysis with alteplase, loading dose dual antiplatelet therapy (DAPT, 100 mg aspirin and 300 mg clopidogrel), single antiplatelet therapy (SAPT), lipid-lowering drugs, and argatroban], and radiological characteristics.

Radiological characteristics encompassed location (such as the internal capsule, basal ganglia, thalamus, pons, lateral ventricle, and centrum semiovale), the culprit vessel supplying the basal ganglia (including the lenticulostriate artery (LSA), posterior choroidal artery, and the recurrent artery of Heubner), stroke subtypes (such as LI and BAD), maximum axial infarct diameter, layers of cerebral infarct lesions, and white matter hyperintensities (WMH). The definition of BAD is based on infarct lesions observed in transversal diffusion-weighted imaging (DWI) scans that extend for at least three consecutive slices within LSA terminations or unilateral involvement of the pons connected to the cerebral surface of the ventral pons without crossing the midline in the paramedian pontine artery (PPA) terminations (1, 18). Maximal axial infarct diameter and layers of cerebral infarct lesions were measured in transversal DWI scans at baseline, with WMH severity at baseline categorized using the modified Fazekas scale (19).

Following data collection, a total of 65 variables were included in the baseline data analysis.

### 2.3 Outcome definition

The primary outcome of this analysis was END, defined as an increase in the NIHSS score of ≥2 and a rise in the motor component of the NIHSS score of ≥1 compared to the initial NIHSS score within 7 days of hospital admission.

### 2.4 Machine learning algorithms

In this study, we utilized seven ML models to predict END in PAI patients: logistic regression (LR) (20), random forest (RF) (21), adaptive boosting (AdaBoost) (22), gradient boosting decision tree (GBDT) (23), histogram-based gradient boosting (HGB) (24, 25), extreme gradient boosting (XGBoost) (26), and category boosting (CatBoost) (27, 28). The best-performing model was selected based on its evaluation metrics. LR, suited for binary classification problems, predicts outcomes by converting a linear function into a sigmoid function, ranging from 0 to 1. RF, an ensemble learning method, combines multiple decision trees, each built from randomly selected subsets of training data and features, to enhance performance and generalizability. AdaBoost, GBDT, HGB, XGBoost, and CatBoost are ensemble learning methods that strengthen models by sequentially training multiple weak learners, with each new model focusing on correcting the errors of its predecessors. The following provides a comprehensive examination of the five ensemble learning methodologies.

#### 2.4.1 AdaBoost

The fundamental principle of the AdaBoost algorithm involves categorizing a collection of weak learners through a process of weighted majority voting, or summation. This method takes into account the errors committed by preceding weak learners and consistently refines the dataset (22, 29).

#### 2.4.2 GBDT

The core concept of GBDT involves employing a gradient-boosting methodology to train a series of decision trees. In each iterative training phase, GBDT computes the residual or gradient of the existing model and utilizes it as the training objective for the subsequent decision tree. The incorporation of new decision trees aims to approximate this residual or gradient, thereby progressively enhancing the performance of the model (23).

#### 2.4.3 HGB

HGB refers to the implementation of gradient boosting algorithms, particularly the popular XGBoost and LightGBM libraries, which use histograms to approximate the distribution of the data. This approach enhances the efficiency and scalability of the model training process without sacrificing much accuracy (24, 25).

XGBoost, a sophisticated and scalable machine learning technique, is renowned for its exceptional proficiency in efficiently managing missing data and seamlessly integrating weak predictive models to form a more precise one. It employs a second-order Taylor expansion to compute the loss function, thereby exhibiting superior performance in both computational speed and prediction accuracy (30, 31).

#### 2.4.4 CatBoost

A machine learning library that has been designed to efficiently handle categorical features. Developed by Yandex, it is renowned for its superior performance in gradient-boosting algorithms, particularly when applied to datasets that contain both numerical and categorical variables (28).

### 2.5 Data processing

In this study, continuous variables were imputed using the median values for each variable to address missing values. Categorical variables were converted into numerical values through dummy encoding. All numerical values were then standardized to ensure uniformity in scale and precision in comparisons.

### 2.6 Feature selection

Feature selection (32), a process used to eliminate superfluous features from a large dataset, improves a machine learning model's efficiency. We employed recursive feature elimination with cross-validation (RFECV), a prominent algorithm in feature selection, which methodically removes the least important features to pinpoint the most effective subset. This study used RFECV based on logistic regression for optimal variable selection.

### 2.7 Model derivation and validation

Patients were randomly divided into training and testing sets at a 7:3 ratio. For model derivation, a shuffle-split cross-validation method was employed to prevent overfitting to a specific dataset and to ensure model generalizability. Shuffle-split cross-validation (33) is a resampling technique employed in machine learning to assess model performance on a constrained data sample. This method entails the random division of the dataset into two subsets: one designated for training and the other for testing. The procedure is executed multiple times, or “folds”, to yield an average performance metric. Widely adopted in machine learning, shuffle-split cross-validation ensures that model efficacy remains robust, avoiding overreliance on specific data partitioning. This approach effectively reduces biases and offers a more dependable estimation of the model's generalization capabilities for novel, unseen datasets.

Additionally, GridSearch CV with shuffle-split cross-validation was utilized to fine-tune and optimize the model hyperparameters on the training set. Supplementary Table 1 details the selected parameter values for each algorithm in the grid-search process. After optimization on the training set, model performance in the testing set was assessed using various metrics, including receiver operating characteristic (ROC) curve, accuracy, F1-score, Matthew's correlation coefficient (MCC), specificity, sensitivity, positive predictive value (PPV), negative predictive value (NPV), and Youden's index.

### 2.8 Model interpretation

To evaluate the importance of each variable, the Shapley Additive exPlanations (SHAP) values were utilized to interpret the machine learning model. Originating from cooperative game theory (34), SHAP assigns an importance value to each feature for a given prediction. A positive SHAP value signifies a beneficial impact on the model's prediction, whereas a negative value indicates an adverse effect. The SHAP method thus serves as a vital tool for understanding and interpreting the behavior of machine learning models.

### 2.9 Statistical analysis

Patients were divided into two groups based on the END outcome: the END group and the clinically stable group. Continuous variables were presented as mean ± standard deviation for normally distributed variables and as medians with interquartile ranges (IQRs) for non-normally distributed data. Categorical variables were expressed as percentages. Categorical variables were analyzed using Fisher's exact test or a χ2 test, while continuous variables were assessed using the Student's *t*-test or Mann–Whitney *U*-test. Statistical analyses were conducted using IBM SPSS Statistics for Windows, version 25.0 software (IBM Corp., Armonk, NY, USA), and a two-sided *p*-value of ≤ 0.05 was considered statistically significant. The ML algorithms were implemented using Python software (version 3.9).

## 3 Results

### 3.1 Baseline characteristics

**Figure 1:**
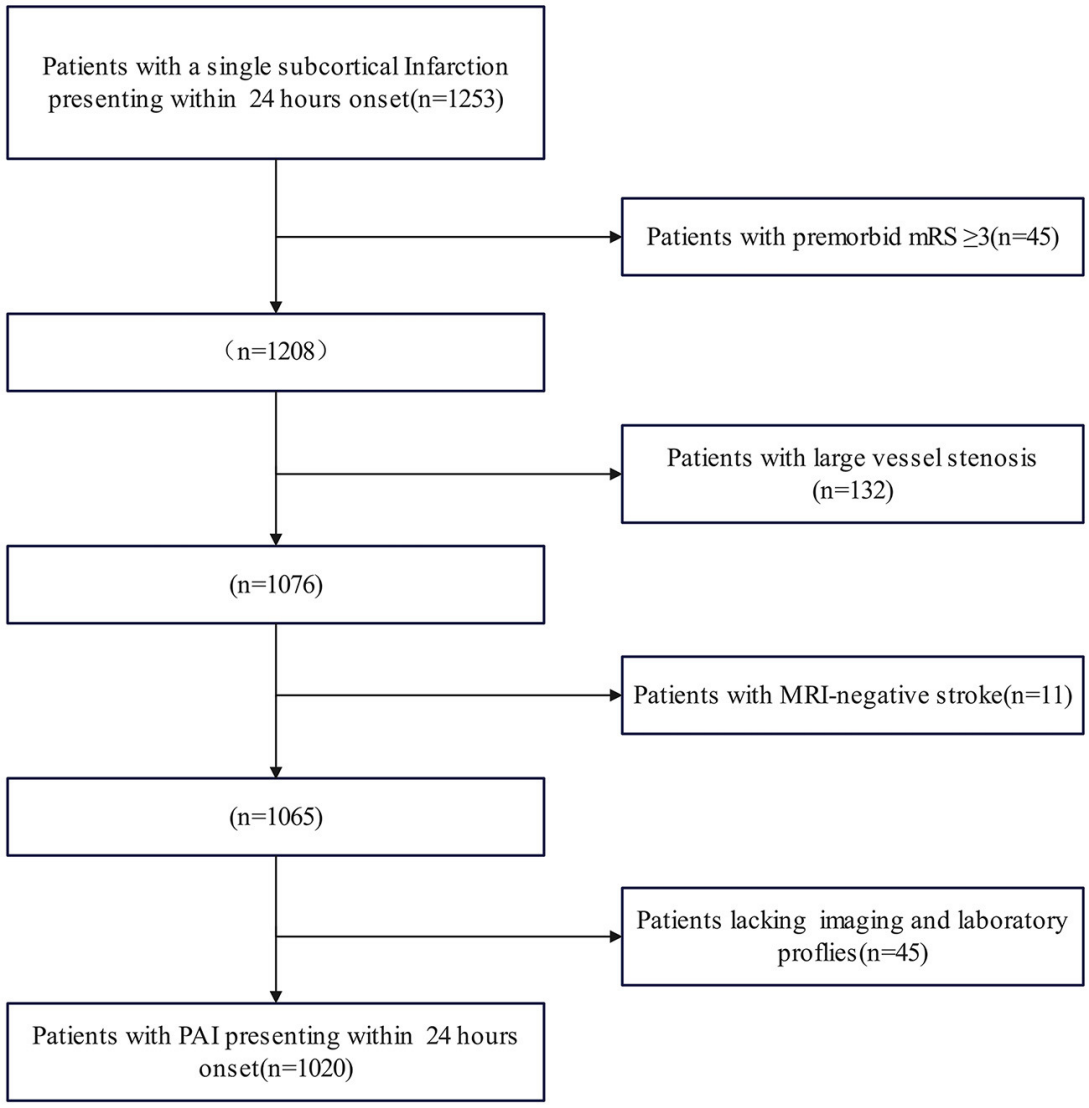
Flow diagram of the included patients.

The study initially included 1,273 patients, but 1,020 PAI patients were enrolled for evaluation after excluding 253 subjects due to missing data and fulfilling the exclusion criteria. Figure 1 presents the patient flow diagram. The baseline characteristics are detailed in Table 1. The average age of the 1,020 patients was 60.46 (range 49.11–71.81) years, with 30.39% of them being women. The END group comprised 129 (12.65%) patients with an average age of 59.08 (range 48.29–69.87) years, and 27.91% were women; the clinically stable group included 891 (87.35%) patients, averaging 60.66 (range 49.24–72.08) years, with 30.74% being women. The median time to END onset was 16 (range 5–25) h. The univariate analysis indicated that factors such as apolipoprotein B (Apo B), the ratio of apolipoprotein A1 (ApoA1) to ApoB, admission NIHSS score, motor arm NIHSS score, motor leg NIHSS score, facial palsy NIHSS score, alteplase, argatroban, lesion location (including the basal ganglia, internal capsule, lateral ventricle, and thalamus), lenticulostriate artery, stroke subtype, and maximal axial infarct diameter were significantly associated with an increased risk of END.

**Table 1:** Baseline variables for the total population and for both groups.

**Variables**	**Clinically stable (*n* = 891)**	**END (*n* = 129)**	**Total (*n* = 1,020)**	***P*-value**
**Demographics**				
Women, *n* (%)	274 (30.74)	36 (27.91)	310 (30.39)	0.5114
Age, mean (SD)	60.66 ± 11.42	59.08 ± 10.79	60.46 ± 11.35	0.1406
BMI, kg/m^2^ (IQR)	24.22 (22.49–26.66)	24.21 (22.82–26.34)	24.22 (22.55–26.66)	0.698
**Medical history** ***n*** **(%)**				
Hypertension	665 (74.63)	97 (75.19)	762 (74.71)	0.8915
Diabetes	165 (18.52)	29 (22.48)	194 (19.02)	0.2839
Coronary heart disease	65 (7.30)	6 (4.65)	71 (6.96)	0.2701
Atrial fibrillation	13 (1.46)	0 (0.00)	13 (1.27)	0.1674
Ischemic stroke	148 (16.61)	16 (12.4)	164 (16.08)	0.224
Hemorrhagic stroke	28 (3.14)	3 (2.32)	31 (3.04)	0.8632
**Secondary prevention treatment**, ***n*** **(%)**				
Antihypertensive treatment	390 (43.77)	46 (35.66)	436 (42.75)	0.0817
Antidiabetic treatment	109 (12.23)	19 (14.73)	128 (12.55)	0.424
Lipid-lowering treatment	150 (16.84)	18 (13.95)	168 (16.47)	0.4069
Antiplatelet therapy	155 (17.40)	21 (16.28)	176 (17.25)	0.7536
Anticoagulant therapy	6 (0.67)	0 (0.00)	6 (0.59)	0.3499
Smoking history, *n* (%)	202 (22.67)	29 (22.48)	231 (22.65)	0.9615
Drinking history (≥20 g/day), *n* (%)	74 (8.31)	13 (10.08)	87 (8.53)	0.5006
**Laboratory data**				
WBC count, ^*^10^9^ (IQR)	6.40 (5.30–7.70)	6.70 (5.49–7.85)	6.43 (5.32–7.70)	0.3883
RBC count, ^*^10^12^ (SD)	4.71 ± 0.56	4.73 ± 0.54	4.75 ± 0.55	0.7484
Hemoglobin, g/L (IQR)	145.00 (136.00–157.00)	146.00 (136.50–157.00)	145.10 (136.00–157.00)	0.4197
Platelet count, ^*^10^12^ (IQR)	255.00 (215.00–312.80)	257.00 (210.00–312.00)	214.50 (175.25–255.00)	0.9621
Lymphocyte count, ^*^10^9^ (IQR)	2.02 (1.54–2.51)	1.97 (1.55–2.50)	1.54 (1.19–2.01)	0.5901
Neutrophil count, ^*^10^9^ (IQR)	5.34 (4.22–6.78)	5.48 (4.41–7.00)	4.23 (3.33–5.38)	0.3002
Neutrophil to lymphocyte ratio (IQR)	2.63 (1.94–3.76)	2.60 (1.88–4.27)	2.62 (1.93–3.84)	0.5424
Platelet-to-lymphocyte ratio (IQR)	138.24 (103.82–177.23)	135.83 (104.29–183.37)	138.23 (104.01–178.40)	0.8353
BUN, mmol/L (IQR)	5.02 (4.19–5.92)	4.66 (3.97–5.95)	4.96 (4.14–5.92)	0.11
Creatinine, μmol/L (IQR)	65.1 (55.2–75.8)	65.2 (53.6–75.55)	65.1 (55–75.78)	0.5847
Glucose on admission, mmol/L (IQR)	6.57 (5.68–8.00)	6.83 (5.8–9.26)	6.59 (5.69–8.10)	0.1123
CRP, mg/L (IQR)	5.35 (3.02–5.80)	5.32 (3.01–5.98)	5.34 (3.02–5.83)	0.5633
D-dimer level on admission mg/L (IQR)	0.10 (0.06–0.16)	0.10 (0.05–0.16)	0.10 (0.06–0.16)	0.6774
TC mmol/L (IQR)	3.91 (3.27–4.58)	4.01 (3.19–4.70)	3.94 (3.26–4.59)	0.5695
Triglyceride, mmol/L (IQR)	1.34 (1.00–1.80)	1.33 (1.01–1.89)	1.34 (1.00–1.80)	0.7775
HDL-C mmol/L (IQR)	0.96 (0.83–1.10)	0.93 (0.82–1.10)	0.96 (0.82–1.10)	0.4494
LDL-C, mmol/L (IQR)	2.41 (1.89–2.95)	2.56 (1.97–3.10)	2.43 (1.89–2.97)	0.1922
ApoA1, mmol/L (IQR)	1.12 (1.04–1.18)	1.12 (1.03–1.20)	1.12 (1.04–1.19)	0.0771
Apo B, mmol/L (IQR)	0.86 (0.78–1.04)	0.96 (0.83–1.09)	0.78 (0.67–0.87)	< 0.0001
ApoA1 to ApoB ratio, (IQR)	1.57 (1.29–1.66)	1.47 (1.14–1.52)	1.57 (1.27–1.65)	0.0001
Homocysteine, μmol/L (IQR)	19.60 (14.30–32.20)	19.00 (15.25–27.25)	19.5 (14.40–31.80)	0.8667
HbA1c, % (IQR)	5.90 (5.60–6.30)	6.00 (5.60–6.42)	5.90 (5.60–6.30)	0.5895
**Clinical features**				
Prestroke mRS score, (IQR)	0 (0–0)	0 (0–0)	0 (0–0)	0.879
Time from onset to presentation, h (IQR)	10 (6–18)	9 (4–15)	10 (5–17)	0.096
Admission NIHSS score, (IQR)	3 (1–4)	4 (2–5)	3 (1–4)	< 0.0001
Motor arm NIHSS score, (IQR)	1 (0–1)	1 (0–2)	1 (0–1)	< 0.0001
Motor leg NIHSS score, (IQR)	1 (0–1)	1 (0–2)	1 (0–1)	< 0.0001
Dysarthria NIHSS score, (IQR)	1 (0–1)	1 (0–1)	1 (0–1)	0.1002
Sensory NIHSS score (IQR)	0 (0–0)	0 (0–0)	0 (0–0)	0.9652
Facial palsy NIHSS score, (IQR)	1 (1)	1 (1)	1 (1)	0.001
Time to onset of END, hours (IQR)	–	16 (5–25)	–	–
**Treatment of the acute phase**, ***n*** **(%)**				
Alteplase	66 (7.41)	17 (13.18)	83 (8.14)	0.0251
Loading DAPT	507 (56.90)	67 (51.94)	574 (56.27)	0.288
SAPT	373 (41.86)	51 (39.53)	424 (41.57)	0.616
Lipid-lowering drugs	877 (98.43)	129 (100)	1,006 (98.63)	0.1517
Argatroban	119 (13.36)	8 (6.20)	127 (12.45)	0.0214
**Lesion location**, ***n*** **(%)**				
Basal ganglia	171 (19.19)	43 (33.33)	214 (20.98)	0.0002
Internal capsule	136 (15.26)	31 (24.03)	167 (16.37)	0.0119
Lateral ventricle	208 (23.34)	18 (13.95)	226 (22.16)	0.0164
Centrum semiovale	70 (7.86)	5 (3.88)	75 (7.35)	0.1055
Pons	143 (16.05)	28 (21.71)	171 (16.76)	0.108
Thalamus	163 (18.29)	4 (3.10)	167 (16.37)	< 0.0001
**Culprit vessel supplying the basal ganglia**, ***n*** **(%)**				
Lenticulostriate artery	507 (56.90)	91 (70.54)	598 (58.63)	0.0033
Posterior choroidal artery	5 (0.56)	0 (0.00)	5 (0.49)	0.3937
The recurrent artery of Heubner	3 (0.34)	1 (0.78)	4 (0.39)	0.4564
**Stroke subtype**, ***n*** **(%)**				
Lacunar infarction	616 (69.14)	44 (34.11)	660 (64.71)	< 0.0001
Branch atheromatous disease	275 (30.86)	85 (65.89)	360 (35.29)	< 0.0001
Maximal axial infarct diameter, *n* (%)				< 0.0001
< 15 mm	684 (76.76)	61 (47.28)	745 (73.04)	
15–20 mm	144 (16.16)	38 (29.46)	182 (17.84)	
>20 mm	63 (7.07)	30 (23.26)	93 (9.12)	
Layers of cerebral infarction lesions, (IQR)	2 (1–3)	2 (2–3)	2 (1–3)	< 0.0001
WMH, (IQR)	2 (1–3)	2 (1–3)	2 (1–3)	0.0513

BMI, body mass index; WBC, white blood cell; RBC, red blood cell; BUN, blood urea nitrogen; CRP, C-reactive protein; TC, total cholesterol; HDL-C, high-density lipoprotein cholesterol; LDL-C, low-density lipoprotein cholesterol; Apo A1, apolipoproteins A1; ApoB, apolipoproteins B; HbA1c, glycated hemoglobin A 1c; mRS, modified Rankin Scale; NIHSS, National Institutes of Health Stroke Scale; DAPT, dual antiplatelet therapy; SAPT, single antiplatelet therapy; WMH, white matter hyperintensities.

### 3.2 Features selection

**Figure 2:**
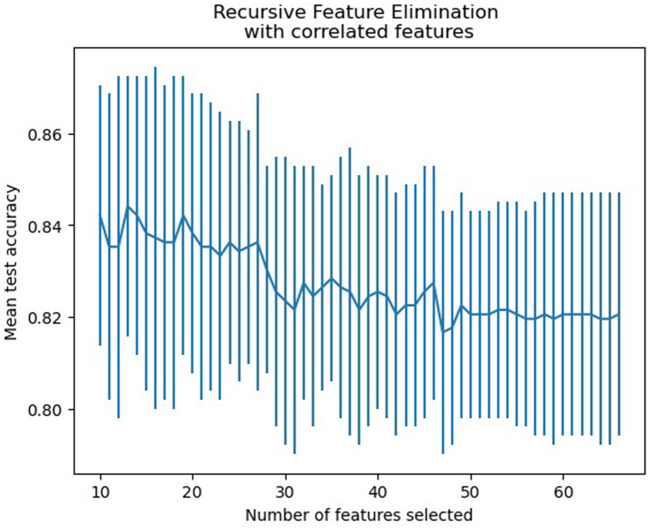
Features selection accuracy curve (the accuracy achieved its peak when the number of variables was 13).

Based on the RFECV results, 13 variables were identified for the predictive model, including blood urea nitrogen (BUN), total cholesterol (TC), low-density lipoprotein cholesterol (LDL-C), apolipoprotein B (apoB), atrial fibrillation, loading-dose dual antiplatelet therapy (DAPT), single antiplatelet therapy (SAPT), argatroban, the basal ganglia, the thalamus, the posterior choroidal arteries, maximum axial infarct diameter (measured at < 15 mm), and stroke subtype. Figure 2 shows how accuracy varies with changes in variables.

### 3.3 Model performance

**Figure 3:**
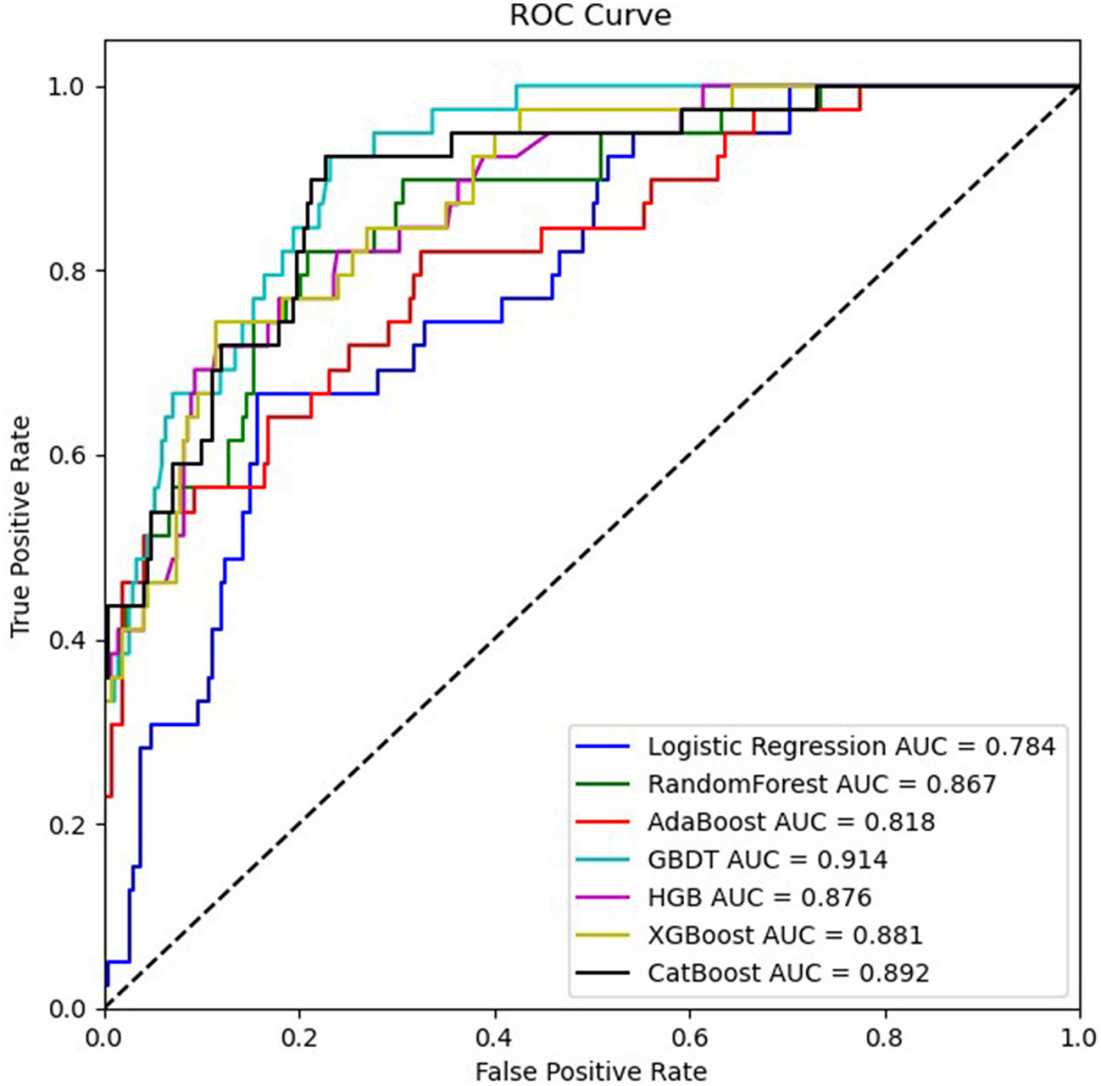
ROC curves of seven ML algorithms based on variables in the testing dataset.

**Figure 4:**
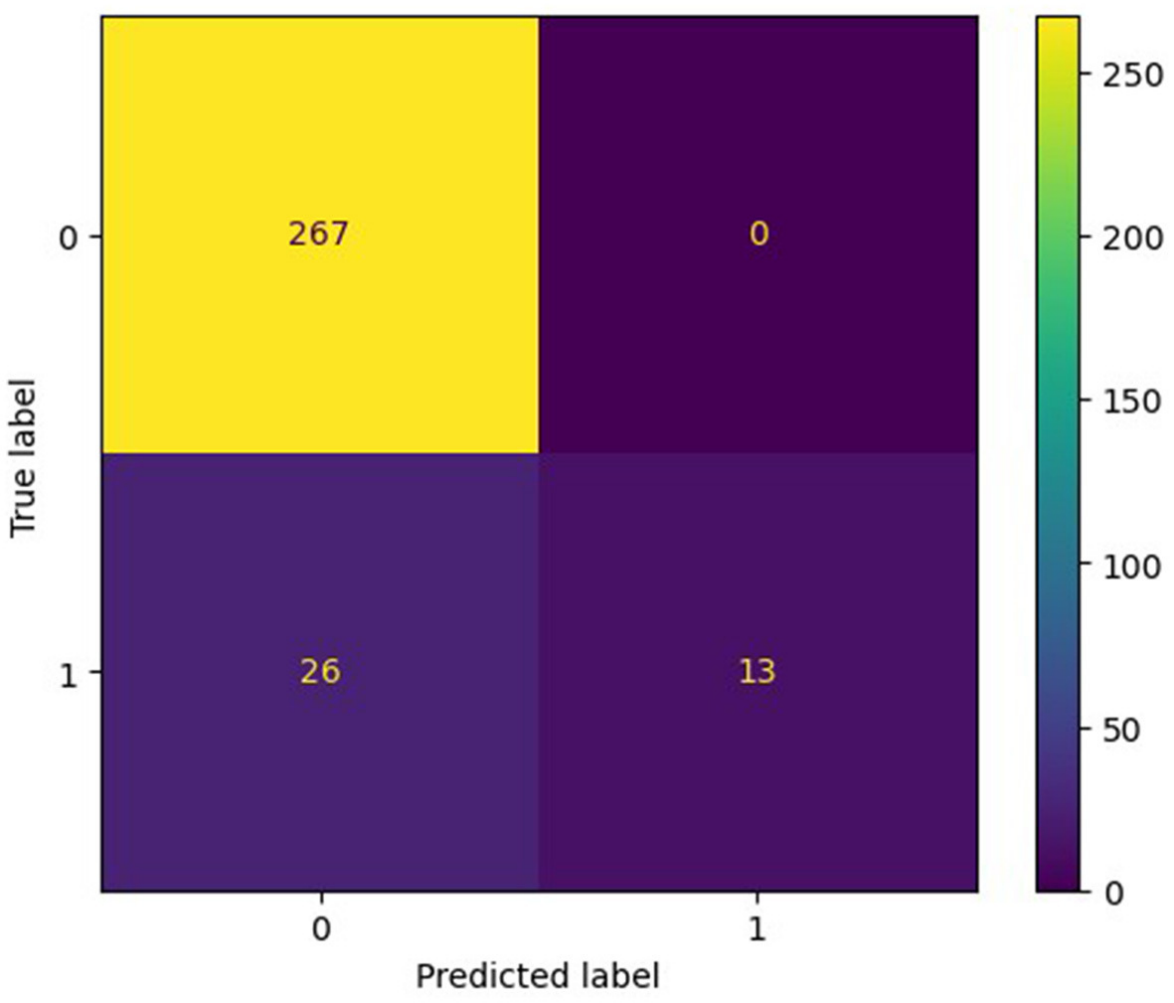
The confusion matrix of the most effective model, GBDT.

The training set for model development comprised 714 patients, including 90 with an END outcome, while the testing set for evaluating model performance consisted of 306 patients, 39 of whom experienced an END outcome. Supplementary Table 2 provides a detailed overview of the features selected for both datasets. We employed seven ML algorithms, namely LR, RF, AdaBoost, GBDT, HGB, XGBoost, and CatBoost, to determine the most effective predictive model. Table 2 displays the area under the curve (AUC) for these seven ML algorithms on both the training and testing datasets. Additionally, it offers a comprehensive analysis of accuracy, F1-score, Matthew's correlation coefficient (MCC), specificity, sensitivity, positive predictive value (PPV), negative predictive value (NPV), and Youden's index for these algorithms on the testing dataset. The GBDT model achieved the highest AUC value at 0.914—an essential measure for evaluating predictive model performance, followed by the CatBoost, XGBoost, HGB, RF, AdaBoost, and LR models (0.8923, 0.8807, 0.876, 0.8639, 0.8184, 0.7838, respectively). Figure 3 illustrates the ROC curve and AUC for each ML classifier in the testing dataset. In conclusion, the GBDT model outperformed the other six ML algorithms, suggesting its superior effectiveness in our study. The confusion matrix for GBDT is shown in Figure 4.

**Table 2:** Summary of prediction results of six ML algorithms based on the training and testing dataset.

	**AUC (95%CI) of the test set**	**AUC (95% CI) of the training set**	**Accuracy**	***F*1-score**	**MCC**	**Specificity**	**Sensitivity**	**PPV**	**NPV**	**Youden's index**
LR	0.7838 (0.6956, 0.8720)	0.7510 (0.6905, 0.8115)	0.8758	0.05	0.1498	1	0.0256	1	0.8754	0.0256
RF	0.8669 (0.7928, 0.9410)	0.9941 (0.9829, 1.005)	0.9118	0.5091	0.5265	0.9965	0.3590	0.8750	0.9138	0.3515
AdaBoost	0.8184 (0.7351, 0.9017)	1 (1, 1)	0.8987	0.4364	0.4385	0.985	0.3077	0.75	0.9069	0.2927
GDBT	0.914 (0.8524, 0.9757)	0.8980 (0.8544, 0.9417)	0.915	0.5	0.5511	1	0.3333	1	0.9113	0.3333
HGB	0.8760 (0.8040, 0.9480)	0.9206 (0.8814, 0.9597)	0.915	0.5	0.5511	1	0.3333	1	0.9113	0.3333
Xgboost	0.8807 (0.8099, 0.9516)	0.9238 (0.8854, 0.9622)	0.915	0.5	0.5511	1	0.3333	1	0.9113	0.3333
CatBoost	0.8923 (0.8243, 0.9602)	0.9550 (0.9248, 0.9851)	0.9085	0.44	0.5052	1	0.282	1	0.905	0.282

### 3.4 Interpretation of the machine learning model

**Figure 5:**
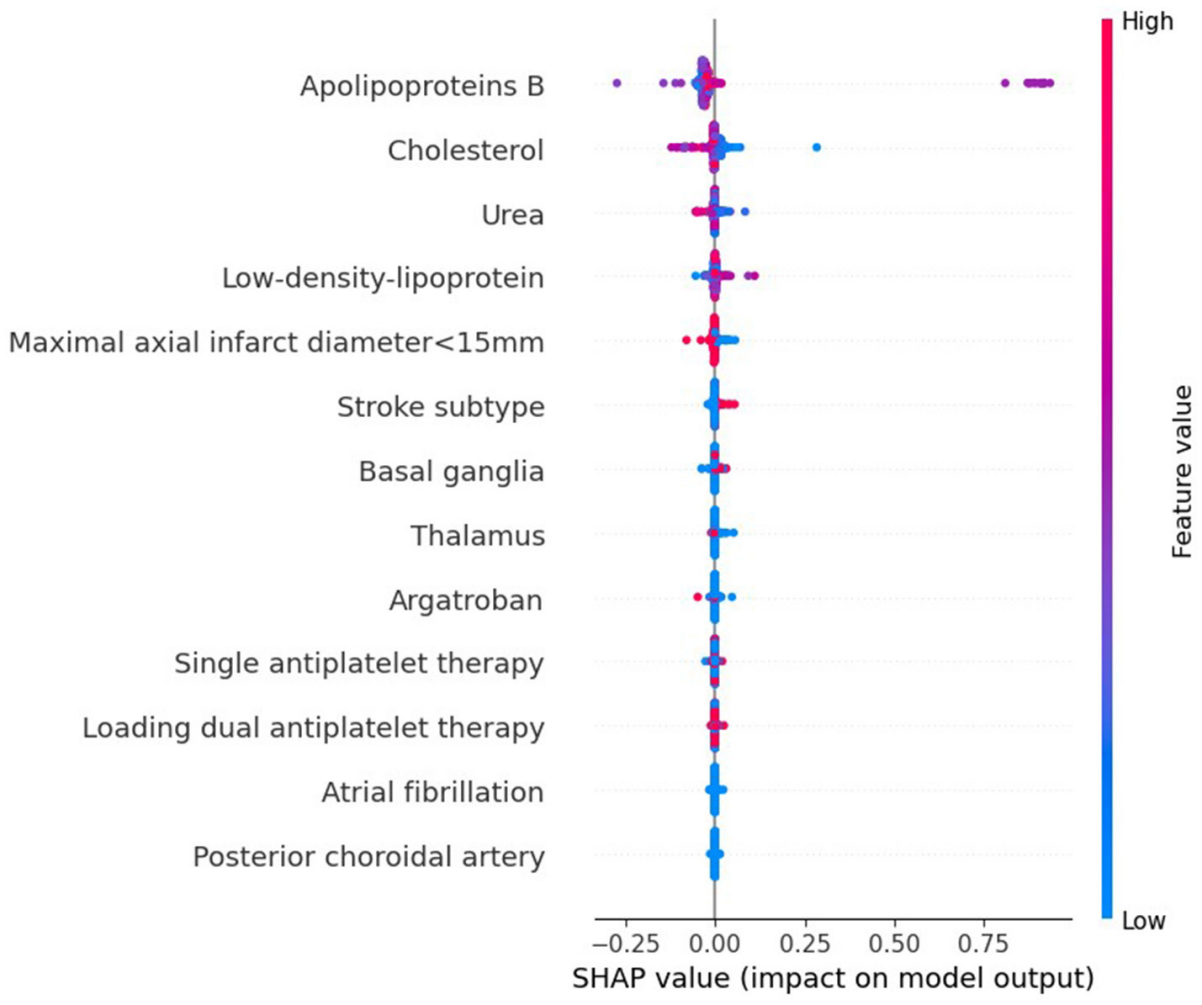
The SHAP summary plot on the test data derived from the optimal prediction model, GBDT.

**Figure 6:**
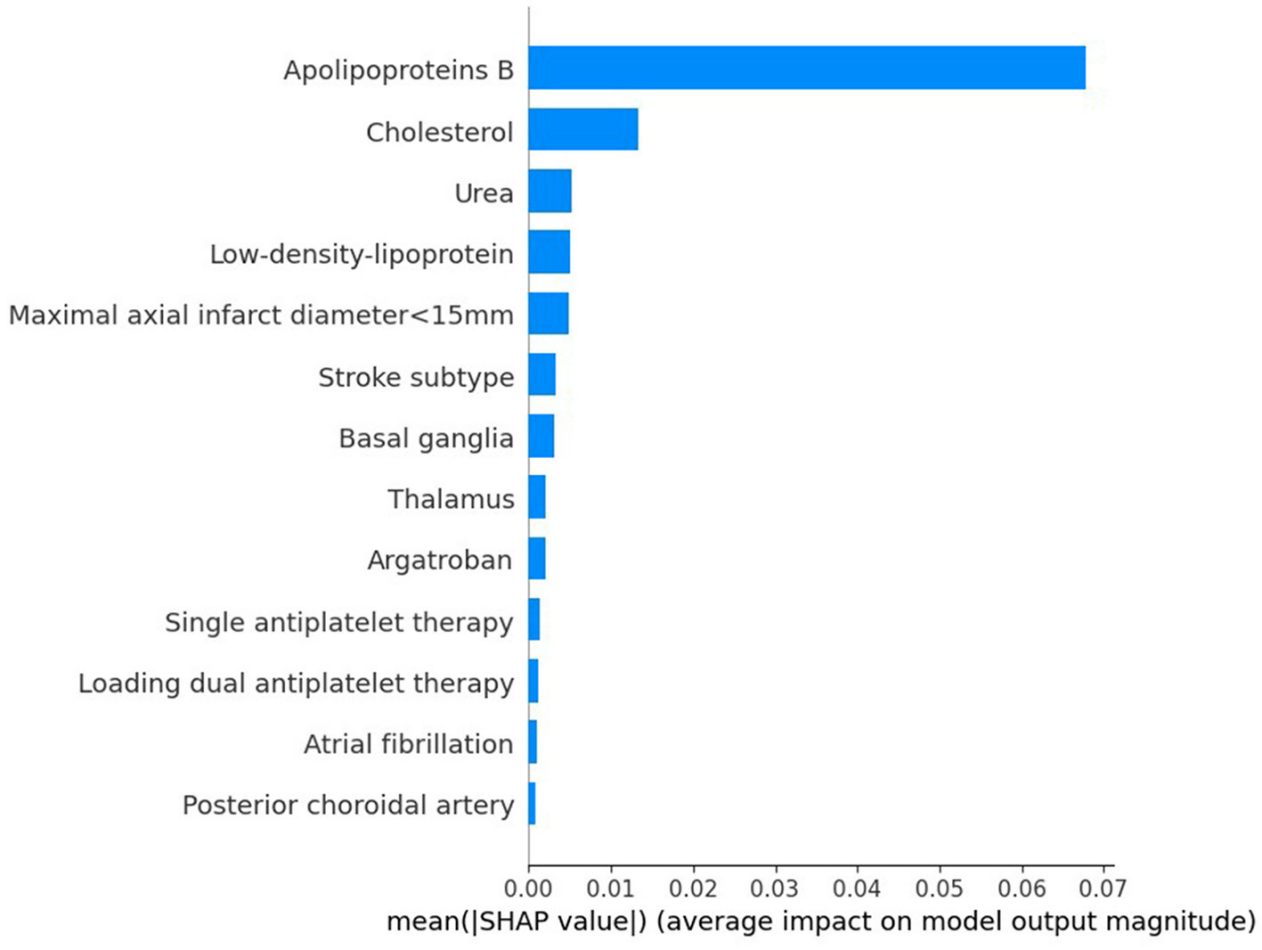
Ranking of the features' importance indicated by SHAP analysis of the best prediction model, GBDT.

To evaluate the significance of each feature in the predictive model, we applied the SHAP method to the GBDT model in the testing dataset. The SHAP value analysis revealed that the most impactful features were apoB, TC, BUN, LDL-C, and a maximal axial infarct diameter of < 15 mm. Following these features were the stroke subtype, the basal ganglia, the thalamus, argatroban, single antiplatelet therapy, and loading dual antiplatelet therapy. Atrial fibrillation and posterior choroidal arteries also contributed to the prediction model but exhibited lower SHAP values. Figure 5 shows the SHAP summary plot for the GBDT model in the testing dataset, where each dot represents an individual case; the color indicates the feature's value (red for higher, blue for lower). Notably, a higher SHAP value for a feature indicates a greater likelihood of END occurrence. Figure 6 displays the ranking of feature importance based on SHAP values.

## 4 Discussion

ML techniques that are integral to artificial intelligence have gained substantial attention and are increasingly employed in medical research for tasks such as screening, diagnosis, and prognosis. Recent studies (15, 35) have investigated the use of ML algorithms in predicting END in patients with acute minor stroke and atrial fibrillation-related stroke. Although these studies yielded promising results with ML, they did not specifically focus on predicting END in patients with PAI—a stroke subtype with a high incidence of END. Our study aimed to use seven ML models to predict END in PAI patients. To address the inherent “black box” nature of ML, we employed the SHAP method to elucidate the predictions of the most effective model, ensuring both the model's performance and its clinical interpretability. This approach enabled the effective communication of information through intuitive visual tools, thereby enhancing clinicians' comprehension of the model's decision-making process and aiding in the clinical application of the prediction results.

In our research, we demonstrated that interpretable machine learning techniques can effectively predict END and personalize predictions for individual patients. The results showed that the GBDT model surpassed six other ML algorithms in terms of AUC and accuracy. Furthermore, the five most important variables associated with END prediction were identified as apoB, TC, BUN, LDL-C, and a maximum axial infarct diameter of < 15 mm. Previous studies have indicated that the END in single subcortical infarctions, including lacunar stroke, is influenced by various factors such as capsular warning syndrome, higher mean arterial pressure at admission, the location of the infarct in the ventral pons, and the extent of hypoperfusion lesion on perfusion-weighted imaging (17). Other factors, such as the initial NIHSS score, pulsatility index, parent artery disease, and neutrophil-to-lymphocyte ratio, also play significant roles (9). In our study, END in PAI was determined by multiple factors, distinguishing it from previous research. The differences between studies may be attributed to variations in study populations, the influence of different statistical methodologies, and the inclusion of diverse variables.

Initially, the END group exhibited dyslipidemia, characterized by higher levels of apoB, TC, and LDL-C compared to the clinically stable group. ApoB, an essential structural component of atherogenic lipoprotein particles such as LDL, lipoprotein, and triglyceride-rich lipoproteins, is acknowledged as a predictor of ischemic cerebrovascular events in patients with preexisting cardiovascular diseases (36). LDL-C, a commonly used clinical lipid marker for assessing lipid-associated risk, including ischemic stroke, has been linked to a reduced frequency of cardiovascular events at lower levels (37). Moreover, previous studies have shown a positive association between TC levels and ischemic stroke risk (38). However, the exact relationship between these lipids and the outcome of END is not yet fully understood. Elevated apoB levels may increase endothelial permeability to LDL, and there is a positive correlation between hypercholesterolemia and apoB generation within plaques. High apoB levels facilitate the penetration of particles into the arterial wall, leading to localized accumulation within the subendothelium. This process heightens the susceptibility to modifications in the artery wall, contributing to the onset of atherosclerosis and plaque progression. Thus, this study suggests that high levels of apoB, TC, and LDL-C, particularly apoB, are linked to END in PAI patients. Previous studies have revealed that a high baseline level of apoB correlates with an increased risk of major adverse cardiovascular events in acute coronary syndrome (39). This study observed lower BUN levels in the END group, with reduced BUN levels correlating with an increased risk of developing END, aligning with previous research (40), which suggested that lower BUN levels were observed in patients with progressive infarction in the anterior circulation and small subcortical infarction.

Previous studies have reported that END is more frequently observed in patients with BAD (41), a trend also evident in our study, suggesting an association between BAD-related PAI and the development of END. We also found that the proportion of maximum axial infarct diameters of < 15 mm was lower in the END group than in the clinically stable group. This finding implies that smaller infarct diameters (< 15 mm), typically linked to LI, are negatively correlated with the occurrence of END. The infarction location significantly influences functional limitations, clinical progression, and patient outcomes (42). Previous studies have indicated that infarct locations within the brainstem, corona radiata, and lenticulostriate artery area, including the internal capsule, are associated with an increased risk of END (4, 43–45). In our study, lesions in the basal ganglia (excluding the internal capsule) and thalamus were identified as predictors of END, with the former located in the lenticulostriate artery area, aligning with previous studies, and the latter showing comparatively favorable outcomes in terms of mortality and permanent motor deficits.

In the management of acute ischemic stroke, antiplatelet therapy is a fundamental therapeutic strategy. Several studies have indicated that DAPT reduces the risk of END (17, 46, 47). In our study, the utilization of antiplatelet therapy in the END group was less frequent than in the clinically stable group. However, our findings suggest that DAPT does not show a clear advantage over SAPT in preventing END. Moreover, we observed that combining argatroban with antiplatelet therapy was associated with a decreased risk of END in patients with PAI, consistent with previous research (48). The contribution of the AF and posterior choroidal artery to the model was found to be minimal.

Our analysis demonstrated that ML models, particularly the GBDT algorithm, showed promising outcomes in predicting END in PAI patients. GBDT, a sophisticated ML algorithm, integrates multiple decision trees to develop a more accurate and robust model. This algorithm effectively handles both continuous and categorical variables, shows a lower susceptibility to overfitting compared to more complex models, and adeptly manages missing data. Considering the inherent multivariate heterogeneity and noise in clinical research data, such as demographic information, laboratory findings, and radiological results available upon hospital arrival, it is critical to choose variables based on prior knowledge. Consequently, we used recursive feature elimination with cross-validation (RFECV) to select 13 predictors for the model, enhancing prediction accuracy.

A strength of this study is that the model performance and clinical interpretability were ensured by using the SHAP algorithm, which was effectively presented to users through easy-to-use visualization tools. Clinicians could better understand the model's decision-making process, thus facilitating the clinical application of prediction results. In addition, our ML model for interpreting predictions was based on a large number of variables, including demographics and laboratory/radiological data obtained from real-world clinical situations at hospital admission. Finally, we demonstrated the potential of interpretable machine learning methods for predicting END in PAI patients and personalizing these predictions within patient populations.

The limitations of our study are as follows: First, the data used in this study is retrospective in nature and sourced from a single center. The retrospective nature of the data may have introduced recall and selection biases to varying degrees. Therefore, it is necessary to use more datasets and conduct prospective multicenter clinical trials to further verify the results and enhance the model's accuracy. Second, we only performed internal validation for dataset validation, but external validation is needed to assess the robustness of the ML model further. Third, missing values were handled using mean interpolation, which inevitably introduces a degree of bias. However, if missing values are removed, some selection bias cannot be completely avoided. Fourth, the exclusion of omics data from the study may potentially limit the predictive performance to some extent.

## 5 Conclusion

We have demonstrated that seven ML models, particularly the GBDT model, can accurately predict END in PAI patients. However, further research with a larger cohort is essential to validating the model's accuracy. Additionally, the predictive efficacy of this model merits exploration in prospective clinical studies.

## Data availability statement

The original contributions presented in the study are included in the article/Supplementary material, further inquiries can be directed to the corresponding authors.

## Ethics statement

The studies involving humans were approved by the Ethics Committee of the Jincheng People's Hospital. The studies were conducted in accordance with the local legislation and institutional requirements. Written informed consent from the patients/participants or patients/participants' legal guardian/next of kin was not required to participate in this study in accordance with the national legislation and the institutional requirements.

## Author contributions

WL: Conceptualization, Data curation, Formal analysis, Funding acquisition, Investigation, Methodology, Project administration, Resources, Software, Supervision, Validation, Visualization, Writing – original draft, Writing – review & editing. LJ: Writing – review & editing. LX: Writing – review & editing. FY: Writing – review & editing. ZG: Writing – review & editing. JL: Writing – review & editing. DZ: Writing – review & editing, Data curation. YL: Data curation, Writing – review & editing. HX: Data curation, Writing – review & editing. HC: Writing – review & editing, Formal analysis. JH: Writing – review & editing. SL: Writing – review & editing. HL: Formal analysis, Writing – review & editing.

## References

[1] Liao X Feng S Wang Y Pan Y Chen W Qu H. Tirofiban combined with aspirin in the treatment of acute penetrating artery territory infarction (STRATEGY): protocol for a multicentre, randomised controlled trial. Stroke Vasc Neurol. (2023) 9:75–81. 10.1136/svn-2022-002284 37220998 PMC10956107

[2] Yaghi S Raz E Yang D Cutting S Mac Grory B Elkind MS. Lacunar stroke: mechanisms and therapeutic implications. J Neurol Neurosurg Psychiatry. (2021) jnnp-2021-326308. 10.1136/jnnp-2021-326308 34039632

[3] Nah H-W Kang D-W Kwon SU Kim JS. Diversity of single small subcortical infarctions according to infarct location and parent artery disease: analysis of indicators for small vessel disease and atherosclerosis. Stroke. (2010) 41:2822–7. 10.1161/STROKEAHA.110.599464 20966406

[4] Jin D Yang J Zhu H Wu Y Liu H Wang Q. Risk factors for early neurologic deterioration in single small subcortical infarction without carrier artery stenosis: predictors at the early stage. BMC Neurol. (2023) 23:83. 10.1186/s12883-023-03128-3 36849878 PMC9969648

[5] Caplan LR. Lacunar infarction and small vessel disease: pathology and pathophysiology. J Stroke. (2015) 17:2–6. 10.5853/jos.2015.17.1.2 25692102 PMC4325635

[6] Regenhardt RW Das AS Lo EH Caplan LR. Advances in understanding the pathophysiology of lacunar stroke: a review. JAMA Neurol. (2018) 75:1273–81. 10.1001/jamaneurol.2018.1073 30167649 PMC7426021

[7] Jeong H-G Kim BJ Yang MH Han M-K Bae H-J. Neuroimaging markers for early neurologic deterioration in single small subcortical infarction. Stroke. (2015) 46:687–91. 10.1161/STROKEAHA.114.007466 25677600

[8] Jiang J Huang X Zhang Y Deng W Shen F Liu J. Total MRI burden of cerebral vessel disease correlates with the progression in patients with acute single small subcortical strokes. Brain Behav. (2019) 9:e01173. 10.1002/brb3.1173 30506998 PMC6346414

[9] Nam K-W Kwon H-M Lee Y-S. Different predictive factors for early neurological deterioration based on the location of single subcortical infarction: early prognosis in single subcortical infarction. Stroke. (2021) 52:3191–8. 10.1161/STROKEAHA.120.032966 34176312

[10] Han X-J. Relationship between lesion patterns of single small infarct and early neurological deterioration in the perforating territory. Eur Rev Med Pharmacol Sci. (2017) 21:3642–8. 28925479

[11] Helleberg BH Ellekjaer H Indredavik B. Outcomes after early neurological deterioration and transitory deterioration in acute ischemic stroke patients. Cerebrovasc Dis. (2016) 42:378–86. 10.1159/000447130 27351585

[12] Chen SD You J Yang XM Gu HQ Huang XY Liu H. Machine learning is an effective method to predict the 90-day prognosis of patients with transient ischemic attack and minor stroke. BMC Med Res Methodol. (2022) 22:195. 10.1186/s12874-022-01672-z 35842606 PMC9287991

[13] Rajkomar A Dean J Kohane I. Machine learning in medicine. N Engl J Med. (2019) 380:1347–58. 10.1056/NEJMra1814259 30943338

[14] Heo J Yoon JG Park H Kim YD Nam HS Heo JH. Machine learning-based model for prediction of outcomes in acute stroke. Stroke. (2019) 50:1263–5. 10.1161/STROKEAHA.118.024293 30890116

[15] Kim S-H Jeon E-T Yu S Oh K Kim CK Song T-J. Interpretable machine learning for early neurological deterioration prediction in atrial fibrillation-related stroke. Sci Rep. (2021) 11:20610. 10.1038/s41598-021-99920-7 34663874 PMC8523653

[16] Wardlaw JM Smith EE Biessels GJ Cordonnier C Fazekas F Frayne R. Neuroimaging standards for research into small vessel disease and its contribution to ageing and neurodegeneration. Lancet Neurol. (2013) 12:822–38. 10.1016/S1474-4422(13)70124-8 23867200 PMC3714437

[17] Vynckier J Maamari B Grunder L Goeldlin MB Meinel TR Kaesmacher J. Early neurologic deterioration in lacunar stroke: clinical and imaging predictors and association with long-term outcome. Neurology. (2021) 97:e143746. 10.1212/WNL.0000000000012661 34400585

[18] Petrone L Nannoni S Del Bene A Palumbo V Inzitari D. Branch atheromatous disease: a clinically meaningful, yet unproven concept. Cerebrovasc Dis. (2016) 41:87–95. 10.1159/000442577 26671513

[19] van Leijsen EMC van Uden IWM Ghafoorian M Bergkamp MI Lohner V Kooijmans ECM. Nonlinear temporal dynamics of cerebral small vessel disease. Neurology. (2017) 89:1569–77. 10.1212/WNL.0000000000004490 28878046 PMC5634663

[20] Nusinovici S Tham YC Chak Yan MY Wei Ting DS Li J Sabanayagam C Wong TY. Logistic regression was as good as machine learning for predicting major chronic diseases. J Clin Epidemiol. (2020) 122:56–69. 10.1016/j.jclinepi.2020.03.002 32169597

[21] Yang L Wu H Jin X Zheng P Hu S Xu X. Study of cardiovascular disease prediction model based on random forest in eastern China. Sci Rep. (2020) 10:5245. 10.1038/s41598-020-62133-5 32251324 PMC7090086

[22] Zheng Z Yang Y. Adaptive boosting for domain adaptation: toward robust predictions in scene segmentation. IEEE Trans Image Process. (2022) 31:5371–82. 10.1109/TIP.2022.3195642 35939457

[23] Seto H Oyama A Kitora S Toki H Yamamoto R Kotoku J. Gradient boosting decision tree becomes more reliable than logistic regression in predicting probability for diabetes with big data. Sci Rep. (2022) 12:15889. 10.1038/s41598-022-20149-z 36220875 PMC9553945

[24] Velichko A Huyut MT Belyaev M Izotov Y Korzun D. Machine learning sensors for diagnosis of COVID-19 disease using routine blood values for internet of things application. Sensors.(2022) 22:7886. 10.3390/s22207886 36298235 PMC9610709

[25] Nhat-Duc H Van-Duc T. Comparison of Histogram-Based Gradient Boosting Classification Machine, Random Forest, and Deep Convolutional Neural Network for Pavement Raveling Severity Classification. Automation in Construction (2023). Available online at: https://www.nstl.gov.cn/paper_detail.html?id=4d53926ab8e76e705f898065f8a78833 (accessed April 19, 2024).

[26] Chen T Guestrin C. XGBoost: a scalable tree boosting system: In: *KDD '16: Proceedings of the 22nd ACM SIGKDD International Conference on Knowledge Discovery and Data Mining*. ACM (2016). 10.1145/2939672.2939785

[27] Dorogush AV Ershov V Gulin A. CatBoost: gradient boosting with categorical features support. arXiv preprint arXiv:1810.11363 (2018). 10.48550/arXiv.1810.11363

[28] Hancock JT Khoshgoftaar TM. CatBoost for big data: an interdisciplinary review. J Big Data. (2020) 7:94. 10.1186/s40537-020-00369-8 33169094 PMC7610170

[29] Tran BX Ha GH Nguyen LH Vu GT Hoang MT Le HT. Studies of novel coronavirus disease 19 (COVID-19) pandemic: a global analysis of literature. Int J Environ Res Public Health. (2020) 17:4095. 10.3390/ijerph17114095 32521776 PMC7312200

[30] Wang R Zhang J Shan B He M Xu J. XGBoost machine learning algorithm for prediction of outcome in aneurysmal subarachnoid hemorrhage. Neuropsychiatr Dis Treat. (2022) 18:659–67. 10.2147/NDT.S349956 35378822 PMC8976557

[31] Hou N Li M He L Xie B Wang L Zhang R. Predicting 30-days mortality for MIMIC-III patients with sepsis-3: a machine learning approach using XGboost. J Transl Med. (2020) 18:462. 10.1186/s12967-020-02620-5 33287854 PMC7720497

[32] Remeseiro B Bolon-Canedo V. A review of feature selection methods in medical applications. Comput Biol Med. (2019) 112:103375. 10.1016/j.compbiomed.2019.103375 31382212

[33] Yu L Zhao Y Wang H Sun T-L Murphy TE Tsui K-L. Assessing elderly's functional balance and mobility via analyzing data from waist-mounted tri-axial wearable accelerometers in timed up and go tests. BMC Med Inform Decis Mak. (2021) 21:108. 10.1186/s12911-021-01463-4 33766011 PMC7995592

[34] Lundberg S Lee SI. A Unified Approach to Interpreting Model Predictions. Nips (2017). Available online at: http://www.xueshufan.com/publication/2618851150 (accessed November 6, 2023).

[35] Sung SM Kang YJ Cho HJ Kim NR Lee SM Choi BK. Prediction of early neurological deterioration in acute minor ischemic stroke by machine learning algorithms. Clin Neurol Neurosurg. (2020) 195:105892. 10.1016/j.clineuro.2020.105892 32416324

[36] Koren-Morag N Goldbourt U Graff E Tanne D. Apolipoproteins B and AI and the risk of ischemic cerebrovascular events in patients with pre-existing atherothrombotic disease. J Neurol Sci. (2008) 270:82–7. 10.1016/j.jns.2008.02.005 18377938

[37] Ntaios G Milionis H. Low-density lipoprotein cholesterol lowering for the prevention of cardiovascular outcomes in patients with ischemic stroke. Int J Stroke. (2019) 14:476–82. 10.1177/1747493019851283 31092149

[38] Zhang Y Tuomilehto J Jousilahti P Wang Y Antikainen R Hu G. Total and high-density lipoprotein cholesterol and stroke risk. Stroke. (2012) 43:646778. 10.1161/STROKEAHA.111.646778 22496337

[39] Hagström E Steg PG Szarek M Bhatt DL Bittner VA Danchin N. Apolipoprotein B, residual cardiovascular risk after acute coronary syndrome, and effects of alirocumab. Circulation. (2022) 146:657–72. 10.1161/CIRCULATIONAHA.121.057807 35770629 PMC9422774

[40] Lin J Mao X Liao Y Luo S Huang Q Song Z. A lesion extending three or more slices as a predictor of progressive infarction in anterior circulation small subcortical infarction. Front Neurol. (2022) 13:926187. 10.3389/fneur.2022.926187 36277920 PMC9579366

[41] Sun S Wang Y Wang Y Men X Bao J Hu X. Lipid and hyperglycemia factors in first-ever penetrating artery infarction, a comparison between different subtypes. Brain Behav. (2017) 7:e00694. 10.1002/brb3.694 28638704 PMC5474702

[42] Yu W Yang J Liu L Song W Zhang Z Xu M. The value of diffusion weighted imaging in predicting the clinical progression of perforator artery cerebral infarction. Neuroimage Clin. (2022) 35:103117. 10.1016/j.nicl.2022.103117 35872435 PMC9421429

[43] Liu H Liu K Zhang K Zong C Yang H Li Y. Early neurological deterioration in patients with acute ischemic stroke: a prospective multicenter cohort study. Ther Adv Neurol Disord. (2023) 16:17562864221147743. 10.1177/17562864221147743 36710721 PMC9880581

[44] Li H Dai Y Wu H Luo L Wei L Zhou L. Predictors of early neurologic deterioration in acute pontine infarction. Stroke. (2020) 51:637–40. 10.1161/STROKEAHA.119.027239 31795900

[45] Berberich A Schneider C Herweh C Hielscher T Reiff T Bendszus M. Risk factors associated with progressive lacunar strokes and benefit from dual antiplatelet therapy. Eur J Neurol. (2020) 27:817–24. 10.1111/ene.14159 31994783

[46] He F Xia C Zhang J-H Li X-Q Zhou Z-H Li F-P. Clopidogrel plus aspirin versus aspirin alone for preventing early neurological deterioration in patients with acute ischemic stroke. J Clin Neurosci. (2015) 22:83–6. 10.1016/j.jocn.2014.05.038 25212871

[47] Yi X Zhou Q Wang C Lin J Chai Z. Aspirin plus clopidogrel may reduce the risk of early neurologic deterioration in ischemic stroke patients carrying CYP2C19^*^2 reduced-function alleles. J Neurol. (2018) 265:2396–403. 10.1007/s00415-018-8998-1 30128710

[48] Xu J Xu X Wang H He L Liu Q Du Y. Dual antiplatelet therapy plus argatroban prevents early neurological deterioration in branch atherosclerosis disease. Stroke. (2022) 53:e19–20. 10.1161/STROKEAHA.121.036356 34784740

