# Supplementary material for: Prediction of early neurologic deterioration in patients with perforating artery territory infarction using machine learning: a retrospective study
Source: Front Neurol. 2024 May 22;15:1368902. doi: 10.3389/fneur.2024.1368902 (PMC11150528; doi:10.3389/fneur.2024.1368902)
Supplement: Supplementary file 1 [file Table_1.DOCX]

***Supplementary Material***

1. **Supplementary Tables**

**Supplementary Table1.** Hyperparameter search settings and final settings.

| **Algorithm** | **Search settings** | **Final settings** |
| --- | --- | --- |
| LR |  |  |
| ‘penalty’ | default | ‘L2’ |
| ‘solver’ | ‘liblinear’ | ‘liblinear’ |
| ‘C’ | [0.1,0.3,0.5,0.8,1.0] | 0.1 |
| RF |  |  |
| ‘criterion’ | default | ‘gini’ |
| ‘n_estimators’ | [50,100,200,300，500,800,1000] | 50 |
| ‘min_samples_split’ | [1,3,5,8,10] | 5 |
| ‘min_samples_leaf’ | [1,3,5,8,10] | 10 |
| ‘max_depth’ | [1,3,5,7,9] | 7 |
| 'max_features’ | [1,3,5,8] | 8 |
| ‘random_state’ | 43 | 43 |
| AdaBoost |  |  |
| ‘learning_rate’ | [0.1,0.5,0.8,1.0,1.2,1.5] | 1.2 |
| ‘n_estimators’ | [50,100,150,200,300,500,800] | 300 |
| ‘max_depth’ | [1,3,5,7] | 3 |
| ‘algorithm’ | default | ’SAMME.R’ |
| GDBT |  |  |
| ‘learning_rate’ | [0.1,0.3,0.5,0.8,1.0] | 0.3 |
| ‘n_estimators’ | [50,100,150,200,300,500,800] | 150 |
| ‘max_depth’ | [1,3,5,7] | 1 |
| ‘min_samples_leaf’ | [10,30,50,80] | 80 |
| ‘min_samples_split’ | default | 2 |
| ‘random_state’ | 43 | 43 |
| HGB |  |  |
| ‘learning_rate’ | [0.1,0.3,0.5,0.8,1.0] | 0.1 |
| 'max_iter' | [20, 50, 80, 100, 120, 150] | 20 |
| ‘max_depth’ | [1,3,5,7] | 1 |
| ‘min_samples_leaf’ | [10,30,50,80] | 10 |
| ‘random_state’ | 43 | 43 |
| Xgboost |  |  |
| ‘learning_rate’ | [0.01,0.05,0.1,0.3,0.5] | 0.01 |
| ‘n_estimators’ | [50,100,150,200,300,500,800] | 300 |
| ‘max_depth’ | [1,3,5,7] | 3 |
| ‘min_child_weight | [0.1,0.3,0.5,0.8,1.0] | 0.5 |
| ‘random_state’ | 43 | 43 |
| ‘gamma’ | default | 0 |
| Catboost |  |  |
| ‘learning_rate’ | [0.01,0.05,0.1,0.3,0.5] | 0.01 |
| ‘iterations’ | [50,100,300,500,800,1000] | 1000 |
| ‘depth’ | [1,3,5,7] | 3 |
| ‘l2_leaf_reg’ | default | 3 |

**Supplementary Table2**. The selected characteristics divided by training data set and testing data set.

| Selected characteristic | Train set (n=714) | Test set (n=306) | Total (n=1020) |
| --- | --- | --- | --- |
| BUN, mmol/L(IQR) | 4.98(4.16,5.92) | 4.96(4.08,5.87) | 4.96(4.14-5.92) |
| TC, mmol/L(IQR) | 3.92(3.26,4.62) | 3.98(3.28,4.49) | 3.94(3.26-4.59) |
| LDL-C, mmol/L(IQR) | 2.46(1.89,2.99) | 2.40(1.91,2.94) | 2.43(1.89-2.97) |
| ApoB, mmol/L(IQR) | 0.78(0.67,0.88) | 0.48(0.68，0.83) | 0.78(0.67-0.87) |
| Atrial fibrillation, n (%) | 11(1.54) | 2(0.65) | 13(1.27) |
| Loading DAPT, n (%) | 396(55.46) | 178(58.17) | 574(56.27) |
| SAPT, n (%) | 304(42.58) | 120(39.21) | 424(41.57) |
| [Argatroban](link:argatroban),n (%) | 93(13.02) | 34(11.11) | 127(12.45) |
| basal ganglia, n (%) | 145(20.31) | 69(22.55) | 214(20.98) |
| Thalamus | 119(16.67) | 48(15.69) | 167(16.37) |
| Posterior choroidal artery | 4(0.56) | 1(0.32) | 5(0.49) |
| Maximal axial infarct diameter ＜15mm, n(%) | 513(71.85) | 232(75.82) | 745(73.04) |
| Stroke subtype, n (%) |  |  |  |
| Lacunar infarction, n (%) | 461(64.57) | 199(65.03) | 660(64.71) |
| Branch atheromatous disease, n (%) | 253(35.43) | 107(34.97) | 360(35.29) |
